# Supplementary material for: Structural and Immunoreactivity Properties of the SARS-CoV-2 Spike Protein upon the Development of an Inactivated Vaccine
Source: Viruses. 2023 Feb 9;15(2):480. doi: 10.3390/v15020480 (PMC9961907; doi:10.3390/v15020480)
Supplement: Supplementary file 1 [file viruses-15-00480-s001.zip › viruses-2135090-supplementary.pdf]

Supplementary material

Figure S1. Infectious activity of virus-containing medium at different time points of inactivation by UV radiation in the absence or presence of riboflavin.

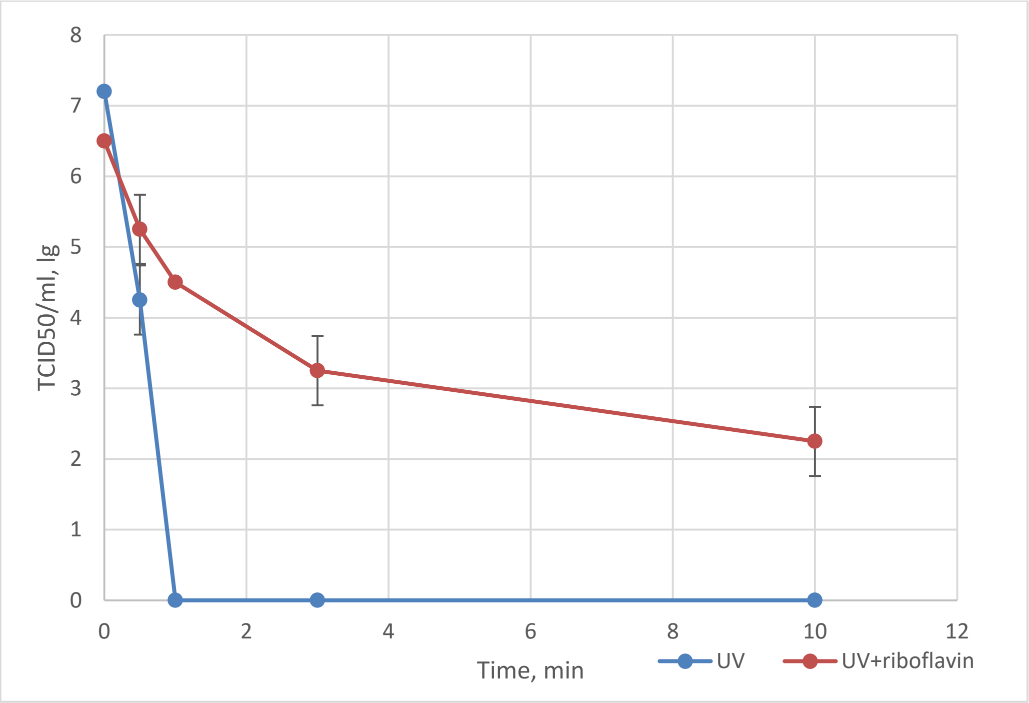

Figure S2. Micrographs of SARS-CoV-2 virions inactivated with β-propiolactone (1:1000, 2 h). On the surface of virions, S-spikes of various morphologies are clearly distinguishable: flails (supposedly pre-fusion conformation) seen in panels A-C and needles (supposedly post-fusion conformation) seen in panels D-F.

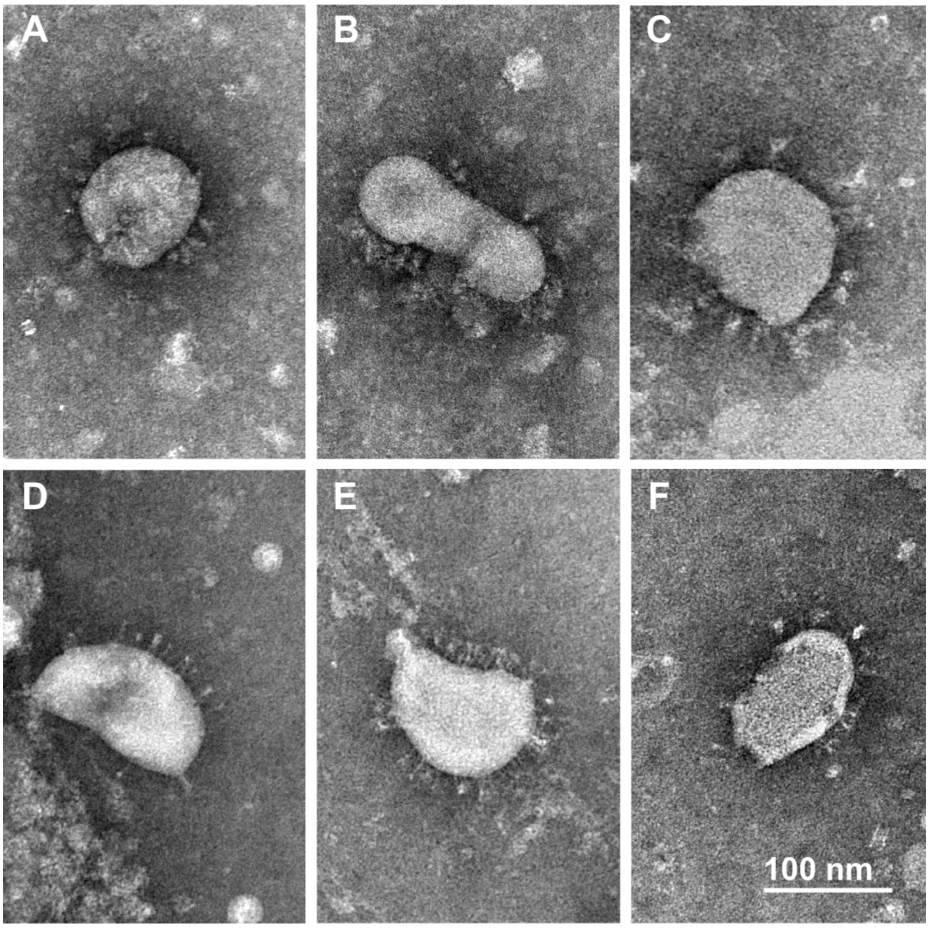

Figure S3. Typical Mascot searches of S-, N-, and M-protein gel bands.

Band S0 (~175 kDa)

Match to: **sp|P0DTC2.1|SPIKE_SARS2** Score: **163** Expect: **3.6e-013**
sp|P0DTC2.1|SPIKE_SARS2 RecName: Full=Spike glycoprotein; Short=S glycoprotein; AltName: Full=E2; AltName: Full=Peplomer protein; Contains: RecName: Full=Spike protein S1; Contains: RecName: Full=Spike protein S2; Contains: RecName: Full=Spike prote

Nominal mass (M_r): **141088**; Calculated pI value: **6.24**
NCBI BLAST search of sp|P0DTC2.1|SPIKE_SARS2 against nr
Unformatted sequence string for pasting into other applications

Variable modifications: Oxidation (M),Propionamide (C)
Cleavage by Trypsin: cuts C-term side of KR unless next residue is P
Number of mass values searched: **53**
Number of mass values matched: **28**
Spike Sequence Coverage: **29%**

Matched peptides shown in **Bold Red**

1 **MFVFLVLLPL VSSQCVNLTT RTQLPPAYTN SFTRGVYYPD K**VFRSSVLHS
51 TQDLFLPFFS NVTWFHAIHV SGTNGTK**RFD NPVLPFNDGV YFASTEK**SNI
101 IRGWIFGTTL DSKTQSLLIV NNATNVVIKV CEFQFCNDPF LGVYYHKNNK
151 **SWMESEFR**VY SSANNCTFEY VSQPFLMDLE GKQGNFKNLR EFVFKNIDGY
201 FKIYSK**HTPI NLVR**DLPQGF SALEPLVDLP IGINITR**FQT LLALHRSYLT**
251 **PGDSSSGWTA GAAAYYVGYL QPR**TFLLKYN ENGTITDAVD CALDPLSETK
301 CTLKSFTVEK **GIYQTSNFRV QPTESIVR**FP NITNLCPFGE VFNATR**FASV**
351 **YAWNR**KRISN CVADYSVLYN SASFSTFKCY GVSPTK**LNDL CFTNVYADSF**
401 **VIR**GDEVRQI APGQTGKIAD YNYKLPDDFT GCVIAWNSNN LDSK**VGGNYN**
451 **YLYR**LFR**KSN LKPFER**DIST EIYQAGSTPC NGVEGFNCYF PLQSYGFQPT
501 NGVGYQPYRV VVLSFELLHA PATVCGPKKS TNLVKNKCVN FNFNGLTGTG
551 VLTESNKK**FL PFQQFGR**DIA DTTDAVRDPQ TLEILDITPC SFGGVSVITP
601 GTNTSNQVAV LYQDVNCTEV PVAIHADQLT PTWR**VYSTGS NVFQTR**AGCL
651 IGAEHVNNSY ECDIPIGAGI CASYQTQTNS PRRARSVASQ SIIAYTMSLG
701 AENSVAYSNN SIAIPTNFTI SVTTEILPVS MTKTSVDCTM YICGDSTECS
751 NLLLQYGSFC TQLNRALTGI AVEQDKNTQE VFAQVKQIYK TPPIKDFGGF
801 NFSQILPDPS KPSKR**SFIED LLFNKVTLAD AGFIKQYGDC LGDIAARDLI**
851 **CAQK**FNGLTV LPPLLTDEMI AQYTSALLAG TITSGWTFGA GAALQIPFAM
901 QMAYR**FNGIG VTQNVLYENQ KLIANQFNSA IGK**IQDSLSS TASALGK**LQD**
951 **VVNQNAQALN TLVKQLSSNF GAISSVLNDI LSRLDKVEAE VQIDR**LITGR
1001 **LQSLQTYVTQ QLIR**AAEIRA SANLAATK**MS ECVLGQSKRV DFCGKGYHLM**
1051 **SFPQSAPHGV VFLHVTYVPA QEK**NFTTAPA ICHDGKAHFP REGVFVSNGT
1101 HWFVTQRNFY EPQIITTDNT FVSGNCDVVI GIVNNTVYDP LQPELDSFKE
1151 ELDKYFKNHT SPDVDLGDIS GINASVVNIQ KEIDRLNEVA KNLNESLIDL
1201 QELGKYEQYI KWPWYIWLGF IAGLIAIVMV TIMLCCMTSC CSCLKGCCSC
1251 GSCCKFDEDD SEPVLKGVKL HYT

Band S1/S2 (~100 kDa)

Match to: **sp|P0DTC2.1|SPIKE_SARS2** Score: **111** Expect: **5.7e-008**
sp|P0DTC2.1|SPIKE_SARS2 RecName: Full=Spike glycoprotein; Short=S glycoprotein; AltName: Full=E2; AltName: Full=Peplomer protein; Contains: RecName: Full=Spike protein S1; Contains: RecName: Full=Spike protein S2; Contains: RecName: Full=Spike prote

Nominal mass (M_r): **141088**; Calculated pI value: **6.24**
NCBI BLAST search of sp|P0DTC2.1|SPIKE_SARS2 against nr
Unformatted sequence string for pasting into other applications

Variable modifications: Oxidation (M),Propionamide (C)

Cleavage by Trypsin: cuts C-term side of KR unless next residue is P
Number of mass values searched: **70**
Number of mass values matched: **23**
Sequence Coverage: **23%**

Matched peptides shown in **Bold Red**

1 **MFVFLVLLPL VSSQCVNLTT RTQLPPAYTN SFTRGVYYPD K**VFRSSVLHS
51 TQDLFLPFFS NVTWFHAIHV SGTNGTKRFD NPVLPFNDGV YFASTEKSNI
101 IRGWIFGTTL DSKTQSLLIV NNATNVVIKV CEFQFCNDPF LGVYYHKNNK
151 **SWMESEFR**VY SSANNCTFEY VSQPFLMDLE GKQGNFKNLR EFVFKNIDGY
201 FKIYSKHTPI NLVRDLPQGF SALEPLVDLP IGINITR**FQT LLALHRSYLT**
251 **PGDSSSGWTA GAAAYYVGYL QPR**TFLLKYN ENGTITDAVD CALDPLSETK
301 CTLKSFTVEK **GIYQTSNFRV QPTESIVR**FP NITNLCPFGE VFNATR**FASV**
351 **YAWNR**KR**ISN CVADYSVLYN SASFSTFK**CY GVSPTKLNDL CFTNVYADSF
401 VIRGDEVRQI APGQTGKIAD YNYKLPDDFT GCVIAWNSNN LDSK**VGGNYN**
451 **YLYR**LFRK**SN LKPFER**DIST EIYQAGSTPC NGVEGFNCYF PLQSYGFQPT
501 NGVGYQPYRV VVLSFELLHA PATVCGPKKS TNLVKNKCVN FNFNGLTGTG
551 VLTESNKK**FL PFQQFGR**DIA DTTDAVRDPQ TLEILDITPC SFGGVSVITP
601 GTNTSNQVAV LYQDVNCTEV PVAIHADQLT PTWR**VYSTGS NVFQTR**AGCL
651 IGAEHVNNSY ECDIPIGAGI CASYQTQTNS PRRARSVASQ SIIAYTMSLG
701 AENSVAYSNN SIAIPTNFTI SVTTEILPVS MTKTSVDCTM YICGDSTECS
751 NLLLQYGSFC TQLNRALTGI AVEQDKNTQE VFAQVKQIYK TPPIKDFGGF
801 NFSQILPDPS KPSKR**SFIED LLFNKVTLAD AGFIK**QYGDC LGDIAARDLI
851 CAQKFNGLTV LPPLLTDEMI AQYTSALLAG TITSGWTFGA GAALQIPFAM
901 QMAYR**FNGIG VTQNVLYENQ KLIANQFNSA IGK**IQDSLSS TASALGK**LQD**
951 **VVNQNAQALN TLVK**QLSSNF GAISSVLNDI LSR**LDKVEAE VQIDR**LITGR
1001 **LQSLQTYVTQ QLIR**AAEIRA SANLAATK**MS ECVLGQSKRV DFCGK**GYHLM
1051 SFPQSAPHGV VFLHVTYVPA QEKNFTTAPA ICHDGKAHFP R**EGVFVSNGT**
1101 **HWFVTQR**NFY EPQIITTDNT FVSGNCDVVI GIVNNTVYDP LQPELDSFKE
1151 ELDKYFKNHT SPDVDLGDIS GINASVVNIQ KEIDRLNEVA KNLNESLIDL
1201 QELGKYEQYI KWPWYIWLGF IAGLIAIVMV TIMLCCMTSC CSCLKGCCSC
1251 GSCCKFDEDD SEPVLKGVKL HYT

Band N (~50 kDa)

Match to: **sp|P0DTC9.1|NCAP_SARS2** Score: **232** Expect: **4.5e-020**
sp|P0DTC9.1|NCAP_SARS2 RecName: Full=Nucleoprotein; Short=N; AltName: Full=Nucleocapsid protein; Short=NC; Short=Protein N

Nominal mass (M_r): **45598;** Calculated pI value: **10.07**
NCBI BLAST search of sp|P0DTC9.1|NCAP_SARS2 against nr
Unformatted sequence string for pasting into other applications

Variable modifications: Oxidation (M),Propionamide (C)
Cleavage by Trypsin: cuts C-term side of KR unless next residue is P
Number of mass values searched: **43**
Number of mass values matched: **24**
Sequence Coverage: **66%**

Matched peptides shown in **Bold Red**

1 MSDNGPQNQR NAPR**ITFGGP SDSTGSNQNG ER**SGARSKQR **RPQGLPNNTA**
51 **SWFTALTQHG KEDLKFPRGQ GVPINTNSSP DDQIGYYR**RA TRRIRGGDGK
101 MKDLSPR**WYF YYLGTGPEAG LPYGANKDGI IWVATEGALN TPK**DHIGTR**N**
151 **PANNAAIVLQ LPQGTTLPKG FYAEGSR**GGS QASSRSSSRS RNSSRNSTPG
201 SSRGTSPAR**M AGNGGDAALA LLLLDR**LNQL ESKMSGK**GQQ QQGQTVTKK**S
251 AAEASKKPRQ KRTATK**AYNV TQAFGRRGPE QTQGNFGDQE LIR**QGTDYK**H**
301 **WPQIAQFAPS ASAFFGMSRI GMEVTPSGTW LTYTGAIKLD DKDPNFKDQV**
351 **ILLNK**HIDAY K**TFPPTEPK**K DKK**KKADETQ ALPQR**QK**KQQ TVTLLPAADL**
401 **DDFSK**QLQQS MSSADSTQA

Bands M + Nfr (~25-30 kDa)*

(1) Match to: **sp|P0DTC9.1|NCAP_SARS2** Score: **108** Expect: **1.1e-007**
sp|P0DTC9.1|NCAP_SARS2 RecName: Full=Nucleoprotein; Short=N; AltName: Full=Nucleocapsid protein; Short=NC; Short=Protein N

Nominal mass (M_r): **45598**; Calculated pI value: **10.07**
NCBI BLAST search of sp|P0DTC9.1|NCAP_SARS2 against nr
Unformatted sequence string for pasting into other applications

Variable modifications: Oxidation (M),Propionamide (C)
Cleavage by Trypsin: cuts C-term side of KR unless next residue is P
Number of mass values searched: **38**
Number of mass values matched: **11**
Sequence Coverage: **42%**

Matched peptides shown in **Bold Red**

1 MSDNGPQNQR NAPR**ITFGGP SDSTGSNQNG ER**SGARSKQR **RPQGLPNNTA**
51 **SWFTALTQHG KEDLK**FPRGQ **GVPINTNSSP DDQIGYYR**RA TRRIRGGDGK
101 MKDLSPR**WYF YYLGTGPEAG LPYGANKDGI IWVATEGALN TPK**DHIGTR**N**
151 **PANNAAIVLQ LPQGTTLPKG FYAEGSR**GGS QASSRSSSRS RNSSRNSTPG
201 SSRGTSPARM AGNGGDAALA LLLLDRLNQL ESKMSGKGQQ QQGQTVTKKS
251 AAEASKKPRQ KRTATK**AYNV TQAFGRRGPE QTQGNFGDQE LIRQGTDYKH**
301 **WPQIAQFAPS ASAFFGMSR**I GMEVTPSGTW LTYTGAIKLD DKDPNFKDQV
351 ILLNKHIDAY KTFPPTEPKK DKKKKADETQ ALPQRQKKQQ TVTLLPAADL
401 DDFSKQLQQS MSSADSTQA

(2) Match to: **sp|P0DTC5.1|VME1_SARS2** Score: **48** Expect: **0.1**
sp|P0DTC5.1|VME1_SARS2 RecName: Full=Membrane protein; Short=M; AltName: Full=E1 glycoprotein; AltName: Full=Matrix glycoprotein; AltName: Full=Membrane glycoprotein

Nominal mass (M_r): **25130**; Calculated pI value: **9.51**
NCBI BLAST search of sp|P0DTC5.1|VME1_SARS2 against nr
Unformatted sequence string for pasting into other applications

Variable modifications: Oxidation (M),Propionamide (C)
Cleavage by Trypsin: cuts C-term side of KR unless next residue is P
Number of mass values searched: **38**
Number of mass values matched: **5**
Sequence Coverage: **33%**

Matched peptides shown in **Bold Red**

1 MADSNGTITV EELKKLLEQW NLVIGFLFLT WICLLQFAYA NRNRFLYIIK
51 LIFLWLLWPV TLACFVLAAV YRINWITGGI AIAMACLVGL MWLSYFIASF
101 RLFARTR**SMW SFNPETNILL NVPLHGTILT RPLLESELVI GAVILR**GHLR
151 IAGHHLGRCD IKDLPKEITV ATSRTLSYYK LGASQR**VAGD SGFAAYSRYR**
201 **IGNYKLNTDH SSSSDNIALL VQ**

*Represented are the best scores found for several analyzed weak bands ~25-30 kDa.

Figure S4. Increase in antibody affinity in paired human sera collected before (day 0) and after (day 42) vaccination with Sputnik V.

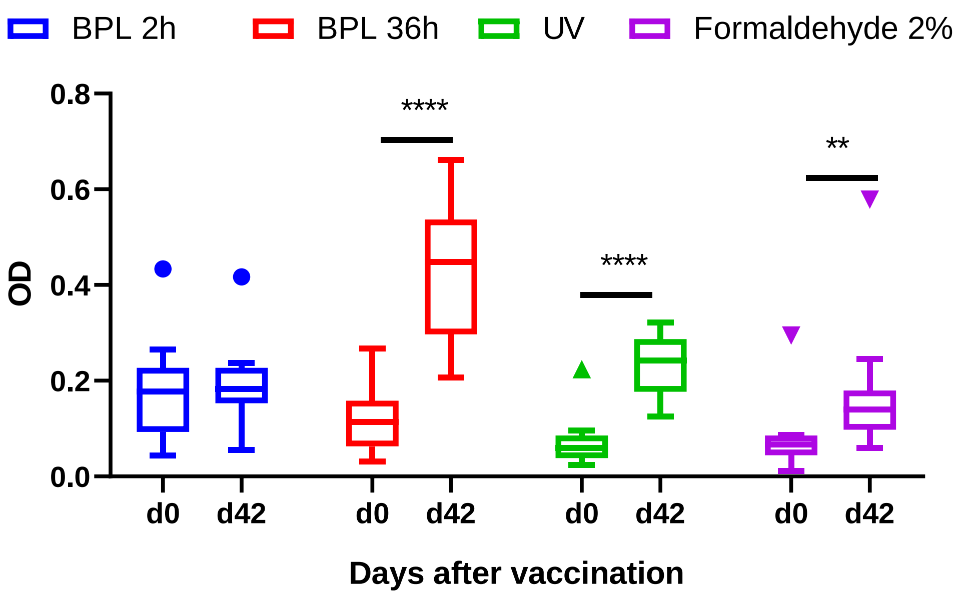

**** p < 0,0001; ** 0,001 < p < 0,01

BPL 2h = β-propiolactone (1:1000, 2h); BPL 36h = β-propiolactone (1:2000, 36h)

Table S1. Neutralization antibody titers of all sera used.*

№	Convalescent	Sputnik V d0	Sputnik V d42	Naïve
1	80	5	320	5
2	80	5	80	5
3	640	5	80	5
4	320	5	40	5
5	40	5	40	5
6	80	5	40	5
7	80	5	160	5
8	20	5	40	5
9	40	5	80	5
10	160	5	40	5
11	160	5	160	-
12	160	5	20	-

*Antibody titers were evaluated by microneutralization assay as described previously [31].
